# Biomaterial-based platforms for in situ dendritic cell programming and their use in antitumor immunotherapy

**DOI:** 10.1186/s40425-019-0716-8

**Published:** 2019-09-04

**Authors:** João Calmeiro, Mylène Carrascal, Célia Gomes, Amílcar Falcão, Maria Teresa Cruz, Bruno Miguel Neves

**Affiliations:** 1 Faculty of Pharmacy, University of Coimbra, 3000-548 Coimbra, Portugal; 2 Center for Neuroscience and Cell Biology, University of Coimbra, 3004-504 Coimbra, Portugal; 3 Tecnimede Group, Sintra, Portugal; 4 Coimbra Institute for Clinical and Biomedical Research, Faculty of Medicine, University of Coimbra, Coimbra, Portugal; 5 Center for Innovation in Biomedicine and Biotechnology, University of Coimbra, Coimbra, Portugal; 6 Coimbra Institute for Biomedical Imaging and Translational Research (CIBIT), University of Coimbra, Coimbra, Portugal; 7 Department of Medical Sciences and Institute of Biomedicine – iBiMED, University of Aveiro, Agra do Crasto - Edifício 30, 3810-193 Aveiro, Portugal

**Keywords:** Biomaterial-based scaffolds, Dendritic cells, In situ mobilization, Antitumor immunotherapy

## Abstract

Dendritic cells (DCs) are central players in the immune system, with an exquisite capacity to initiate and modulate immune responses. These functional characteristics have led to intense research on the development of DC-based immunotherapies, particularly for oncologic diseases. During recent decades, DC-based vaccines have generated very promising results in animal studies, and more than 300 clinical assays have demonstrated the safety profile of this approach. However, clinical data are inconsistent, and clear evidence of meaningful efficacy is still lacking. One of the reasons for this lack of evidence is the limited functional abilities of the used ex vivo-differentiated DCs. Therefore, alternative approaches for targeting and modulating endogenous DC subpopulations have emerged as an attractive concept. Here, we sought to revise the evolution of several strategies for the in situ mobilization and modulation of DCs. The first approaches using chemokine-secreting irradiated tumor cells are addressed, and special attention is given to the cutting-edge injectable bioengineered platforms, programmed to release chemoattractants, tumor antigens and DC maturating agents. Finally, we discuss how our increasing knowledge of DC biology, the use of neoantigens and their combination with immune checkpoint inhibitors can leverage the refinement of these polymeric vaccines to boost their antitumor efficacy.

## Dendritic cell-based approaches in antitumor immunotherapy

Approaches to enhance or restore the immune system aptitude to identify and destroy malignant cells have long been viewed as a central goal in cancer treatment [1–3]. The use of dendritic cells (DCs), powerful modulators of immune responses, in immunotherapy has been extensively scrutinized and has been highly desirable for clinical application since the early 1990s. There are more than 300 completed or ongoing registered clinical trials using these cells as antitumor vaccines [4]. Currently, there are mainly two approaches for exploring DCs in oncologic treatments: 1) vaccines constituted by ex vivo-generated DCs matured and loaded with tumor antigens and 2) in vivo direct targeting of antigens to DCs [5]. Manipulation of DCs ex vivo followed by their injection back into the patient is the most common approach, which is being used in 97% of referenced clinical trials [4]. In this approach, blood precursors (CD14^+^ monocytes or CD34^+^ hematopoietic stem cells) are collected from patients, differentiated into DCs, loaded with antigens and matured. The resultant cellular product is cryopreserved and then released for administration according to the defined vaccination schedule.

These types of vaccines present exceptional tolerability, but the procedure is highly expensive and laborious as result of the required manipulation in GMP conditions and notwithstanding the good safety profile, the rate of success is inconsistent [4]. In fact, objective tumor responses using standard oncologic criteria are usually low, with reports ranging from 3.3 to 15% [6–8]. Furthermore, promising vaccines in early phase studies [9–12] often fail to present clear beneficial clinical outputs in phase III trials [13]. So far, only sipuleucel-T, an autologous antigen-presenting cell vaccine for the treatment of asymptomatic metastatic hormone refractory prostate cancer, has demonstrated satisfactory efficacy in phase III trials and was approved by the Food and Drug Administration (FDA) in 2010. The lack of robustness of DC-antitumor immunotherapies was attributed in part to low numbers of injected cells that are able to migrate to the lymph nodes and to prime T lymphocytes [14, 15] and also to functional limitations of the ex vivo*-*differentiated DCs. These DCs, which are differentiated from hematopoietic precursors, have been shown to be less efficient than endogenous DC subpopulations, specifically in their competence to cross-present antigens to CD8^+^ T cells [16, 17]. The lack of definition of immunogenic neoantigens, the use of shared antigens, the induction of low levels of CD8^+^ T cell responses and the inexistence of standardized production and manufacturing protocols are other reasons to explain the poor efficacy of DC vaccines.

To overcome the limitations of ex vivo manipulated DC vaccines, several strategies aiming to directly target antigens to endogenous DCs have been developed in recent years [18, 19]. These strategies encompass antigen coupling to monoclonal antibodies specific to DC surface molecules, including XCR1, DCIR, Cleac9A, CD40, DC-SIGN DEC-205 and the mannose receptor. Preclinical and clinical studies demonstrated encouraging results, with the establishment of effective antitumor CD8^+^ and CD4^+^ T cell responses and humoral immunity [20–28]. However, clinical implementation has been struggling with several challenges: the approach demands the co-administration of DC maturation agents; otherwise, it is prone to induce tolerance to the vehiculated antigen [29]; it is limited to immunization with one known tumor antigen at a time; and the targeted receptor needs to be unequivocally expressed by the selected DC subpopulation.

Another way to explore the immunogenic power of endogenous DC populations in cancer therapies relies on strategies for their in situ mobilization and modulation. They consist of implantable or injectable biomaterial-based scaffolds providing a specific microenvironment that allows the recruitment of desired DC populations and potentiates their interaction with other immune effectors. Seminal and promising applications of this approach, which encompass both biotechnology and immunology, have gradually appeared in the cancer immunotherapy field and will be the focus of the present review.

## Strategies for in situ DC mobilization and antigen loading

### GM-CSF-secreting tumor cells

One of the first approaches used for in situ mobilization and activation of endogenous DCs was the use of irradiated tumor cells that were genetically altered to secrete cytokines/chemokines [30, 31]. Among these strategies, GM-CSF-secreting tumor cell vaccines attracted particular interest [32]. GM-CSF is a hematopoietic cytokine with multiple effects on the immune system: it directly influences hematopoiesis and expansion of granulocytes, macrophages, DCs, eosinophils and neutrophils [33, 34] and indirectly modulates T cell activation and proliferation [35]. In the context of DC-based antitumor vaccines, GM-CSF is particularly appealing, given that it is a powerful DC chemoattractant and a maturation inducer [36–38]. Furthermore, GM-CSF also presents immune-independent effects by directly inhibiting cancer cell proliferation [39, 40].

Seminal studies by Glenn Dranoff and colleagues, performed with the B16 melanoma mouse model, demonstrated that intradermal injection of irradiated GM-CSF-secreting tumor cells efficiently induces strong, specific and prolonged antitumor immunity [30]. The main action of the approach is due to the generation of a local inflammatory reaction with recruitment and activation of DCs, macrophages and granulocytes [30, 41–43]. Briefly, GM-CSF secreted by modified tumor cells attracts DCs to the injection site. Recruited DCs engulf apoptotic tumor cells and mature via the effect of released GM-CSF. Then, mature DCs migrate to draining lymph nodes to efficiently present processed tumor antigens to T cells, resulting in lymphocyte activation and expansion with the consequent boost of the antitumor immune response. Clinically, several phase I/II clinical trials exploring this type of vaccine have shown a coherent induction of humoral and cellular immunity in several cancers, such as melanoma [44, 45]; pancreatic [46–48], prostate [49, 50], kidney [51] cancer; and chronic myeloid leukemia [52].

However, these vaccines present some drawbacks. The sustained GM-CSF release by injected tumor cells can paradoxically lead to disease progression due to the provocation of immune tolerance via the differentiation of tolerogenic DCs and the recruitment of myeloid suppressor cells [53–55]. Moreover, clinical trial outcomes are often variable, with tumor regressions being inconsistent within patients and with phase III trials that continuously failed [32, 56]. Hence, despite initial promising results, the GVAX vaccine - a whole cell pancreatic cancer vaccine plus GM-CSF-expressing tumor cells - failed due to lack of efficacy [57]. However, we are currently in an exciting era of scientific achievements in cancer immunotherapy, supported by a growing knowledge on the precise interactions of tumors and the different immune players. Thus, new vaccine designs accommodating this information and exploring novel biotechnological solutions are required and highly anticipated.

### Biomaterial-based platforms for DC recruitment and antigen loading

Biomaterial-based nanosized delivery systems, including polymeric nanoparticles, dendrimers and liposomes, have long been viewed as a valuable approach to enhance antitumor immunity (reviewed in [58]). These nanoparticles carry immunomodulatory agents and tumor antigens and, after capture by host DCs, elicit strong immune responses. [59, 60]. In a preclinical context, the approach was efficient for some types of cancer; however, clinical translation faces several challenges. There is some risk of off-target effects, systemic cytotoxicity, problems related to stability, cargo bioavailability and long-term efficacy.

**Fig. 1:**
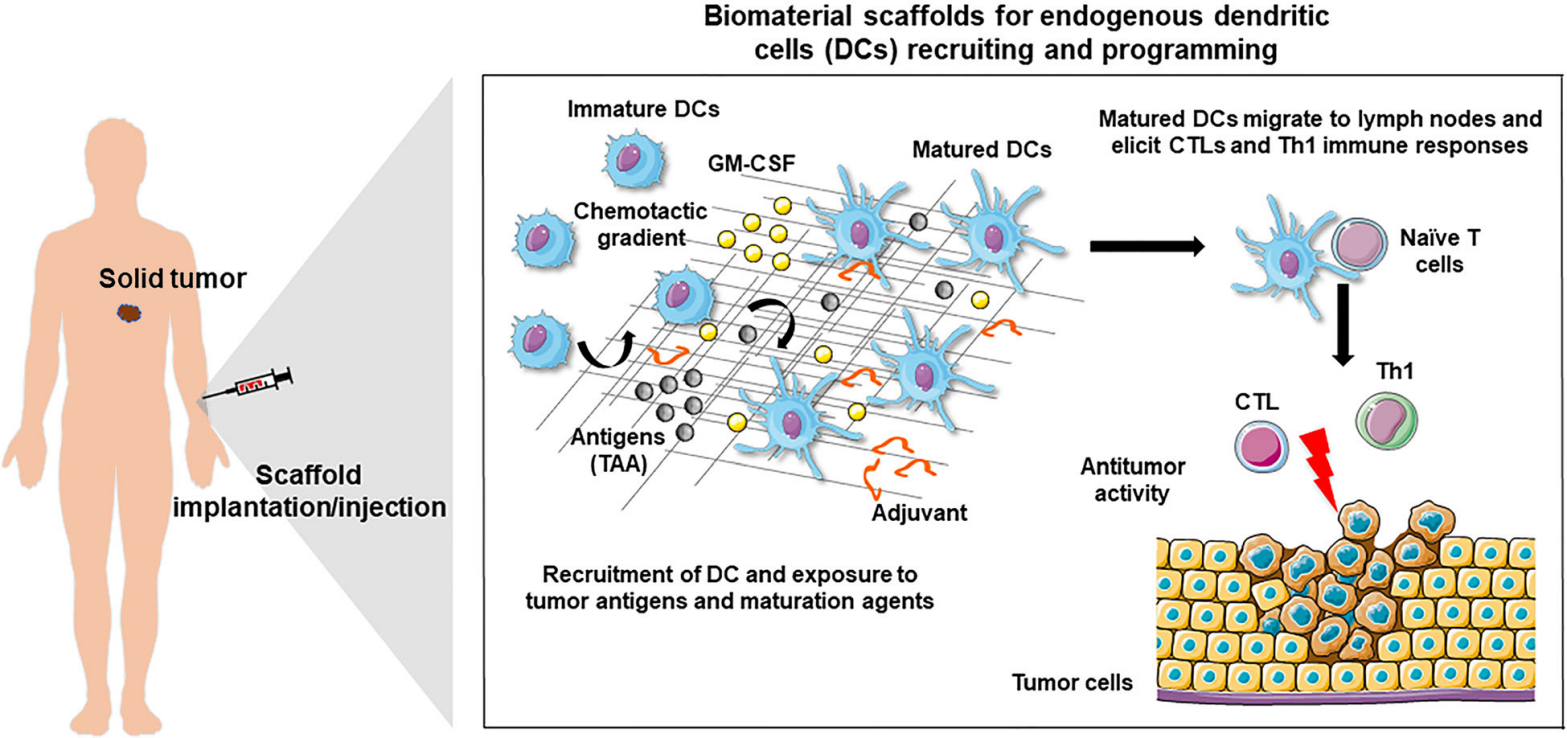
Biomaterial-based scaffold application in DC recruitment and programming for enhanced antitumor activity. A subcutaneously implanted or injected biocompatible polymer scaffold is designed to include and release, in a controlled way, a DC chemotactic agent, an adjuvant, and a source of tumor antigens. The loaded chemoattractant, e.g., GM-CSF, recruits immature dendritic cells (iDCs) into the macroporous matrix where they are exposed to adjuvants and tumor antigens. Mature antigen-loaded DCs (matured DCs) then migrate out of the scaffold to the lymph nodes, presenting processed antigens to T-cells, and boosting antitumor immunity that way

In 2002, Tadashi Kumamoto and collaborators conceived a novel strategy to modulate endogenous DCs envisaging a specific immune response. They resorted to subcutaneous implantation of a biomaterial-based scaffold designed to release DC chemoattractants alongside the tumor lysate [61]. Endogenous DCs are recruited to the scaffold where they are fueled and activated by released antigens and maturating agents, respectively. The rationale is similar to using tumor cells modified to release chemokines; however, it allows for the precise control of the release of chemoattractants, antigens and maturation inducers. Furthermore, these 3D matrices work as platforms that favor the interaction between DCs and additional immune cells, such as T and NK cells (Fig. 1**)**.

In the last decade, this concept of biomaterial-based DC programming systems has gained significant relevance, with the emergence of two different strategies: two-step or one-step approaches, depending on the time of loading of antigens (Table 1).

**Table 1:** Overview of different existing anti-cancer biomaterial-based vaccines for DC recruitment and antigen loading

Approach	Scaffold biomaterial	Load	Administration	Target/tumor model
Two step	Ethylene-vinyl-acetate (EVA) polymers rods	1st CCL19 2nd Tumor lysate	Coimplantation	E.G7-OVA tumor cells injected mice [61]
	Hydrogel - Thermosensitive monomethoxypoly(ethylene glycol)-*co*-poly(lactic-*co*-glycolic acid) copolymer (mPEG-PLGA)	1st - GM-CSF 2nd - Tumor antigens	2 injections (injection of viral or nonviral vectors in a 2nd step)	Murine melanoma model [62]
One step	Poly(lactide-co-glycolide)	GM-CSF, CpG, autologous tumor lysate	Implantation	Human melanoma Phase I clinical trial NCT01753089
	Poly(lactide-co-glycolide)	GM-CSF, CpG, tumor lysate	Implantation	Murine melanoma model [41, 63–66]
	Poly(lactide-co-glycolide)	GM-CSF, CpG, tumor lysate	Implantation	Murine Lewis lung carcinoma (LLC) [64]
	Poly(lactide-co-glycolide)	GM-CSF, CpG, tumor lysate	Implantation	Rat glioma model [67, 68]
	Poly(lactide-co-glycolide)	GM-CSF, CpG, Tumor lysate	Implantation	Murine melanoma model; combination with anti PD-1 or CTLA-4 mAb [69]
	Poly(lactide-co-glycolide)	CCL20, CpG, tumor lysate	Implantation	Murine melanoma model [66]
	Poly(lactide-co-glycolide)	Flt3L, CpG, tumor lysate	Implantation	Murine melanoma model [66]
	Poly(lactide-co-glycolide)	GM-CSF, MPLA, tumor lysate	Implantation	Murine melanoma model [64]
	Poly(lactide-co-glycolide)	GM-CSF, Poly-I:C, Tumor lysate	Implantation	Murine melanoma model [64]
	Poly(lactide-co-glycolide)	GM-CSF, Poly-I:C, tumor lysate	Implantation	Murine Lewis lung carcinoma (LLC) [64]
	Hydrogel/cryogel–alginate polymer	GM-CSF, CpG, irradiated tumor cells	Injection	Murine breast cancer [70]
	Hydrogel/cryogel–alginate polymer	GM-CSF, CpG, irradiated tumor cells	Injection	Murine melanoma model [71]
	Covalent and ionic crosslinked cryogel–alginate polymer	GM-CSF, CpG, irradiated -tumor cells	Injection	Murine breast cancer [72]
	Crosslinking hydrogel- dextran vinylsulfone and tetra-thiolated polyethyleneglycol	CCL20 + PLGA microparticles encapsulating IL-10, siRNA and DNA antigen	Injection	Murine A20 B cell lymphoma [73]
	Mesoporous silica rods (MSRs) - synthetic amorphous silica	GM-CSF, CpG, OVA	Injection	Prophylactic action in a murine model, injected with EG7-OVA lymphoma cells [74]

### Two-step approach

In the seminal work of Tadashi Kumamoto, ethylene-vinyl-acetate (EVA) polymer rods releasing chemokine (C-C motif) ligand 19 (CCL19) were subcutaneously implanted in the abdominal skin of mice [61]. More than 70% of the chemokine was released in a fully functional form in the first 48 h. This resulted in the recruitment and transitory entrapment of Langerhans cells (LCs), a particular subset of skin DCs, into the scaffold. Antigen loading was achieved in a second step by the (co)implantation of EVA rods carrying tumor lysates, defined MHC I-restricted peptides or artificial xenogeneic antigens. To trigger maturation and LC migration from the epidermis to draining lymph nodes, haptens such as DNFB or oxazolone were applied over the implantation sites. The strategy was as effective as conventional ex vivo DC vaccines in eliciting tumor-specific Cytotoxic T-lymphocyte (CTL) activities. Moreover, the authors demonstrated the efficacy of the approach in fibrosarcoma, E. G7-OVA tumor and Lewis lung carcinoma mouse models, both in a prophylactic (implantation of rods before tumor inoculation) and therapeutic (implantation of rods after tumor inoculation) context [61].

Following a similar strategy, a novel and more advanced two-step approach based on hydrogel matrices was developed [62]. First, DCs are attracted to an injectable thermosensitive monomethoxypoly(ethylene glycol)-*co*-poly(lactic-*co*-glycolic acid) copolymer (mPEG-PLGA) hydrogel via continuous and controlled release of GM-CSF [62, 75]. In a second phase, viral and nonviral vectors were used to deliver cancer antigens and to program recruited DCs. The hydrogel scaffold was able to release GM-CSF and recruit DCs and macrophages. This strategy resulted in the production of strong tumor-specific immune responses in therapeutic and prophylactic settings of murine melanoma models [62].

### One-step approach

#### Implantable structures

As an evolution of the two-step system, in the last decade, David Mooney and collaborators conceived several biomaterial-based implantable or injectable platforms for endogenous DC recruitment and antigen loading, all in a single step. Biocompatible polymers were designed to include and release, in a controlled way, a DC chemotactic agent, adjuvants and tumor antigens [63]. Several of these approaches are based on an extremely porous scaffold composed of poly(lactide-coglycolide) (PLG). PLG has multiple applications in the biomedical field owing to its specific characteristics: FDA approved for clinical use, prone to surface modification to enhance biological interactions, high biocompatibility and tailorable biodegradation rate [76].

Using a high-pressure CO_2_ foaming process, GM-CSF was encapsulated into macroporous PLG matrices with efficiencies above 50% [77, 78]. These scaffolds release up to 60% of loaded GM-CSF during the initial 5 days, with the remaining gradually released during an additional 10 days [63]. To strongly activate recruited DCs, CpG-oligonucleotides (CpG-ODN) were also immobilized to the matrices. For this, CpG-ODNs were condensed with polyethylenimine to form cationic nanoparticles that electrostatically interact with the anionic PLG biomaterial, resulting in a retention higher than 80% over 25 days [63]. The scaffolds containing GM-CSF, melanoma tumor lysates and CpG-ODN were assayed in the syngeneic B16-F10 murine melanoma model across several works. The structures were able to attract and activate several DC subsets (CD11c^+^, pDCs and CD8^+^ DCs) for at least 2 weeks [65]. Importantly, the number of DCs accumulated in the scaffold was of the same magnitude as that commonly administered in ex vivo-generated DC protocols [63]. Vaccination with these 3D macroporous structures elicited robust tumor-specific CTL responses promoting complete tumor regression in 47% of mice [41], 50% survival in a therapeutic goal, 33% in a long-term survival goal and a notable 90% in a prophylactic goal [63, 64].

In subsequent studies, PLG matrices were used to supply other chemokines, such as CCL20 and Flt3L, or other adjuvants, such as MPLA and Poly-I:C, ligands for TLR4 and TLR3, respectively [64, 66]. Disregarding the adjuvant used, vaccine efficacy was shown to highly correlate to the quantities of recruited CD8^+^ and pDCs alongside local GM-CSF and IL-12p70 concentrations [64]. PLG scaffolds were also tested in combination with monoclonal antibodies, targeting the immune checkpoints programmed cell death ligand 1 (PD-L1) and cytotoxic T-lymphocyte antigen 4 (CTLA-4). These combinations elicited strong CTL activity and tumor regression, reaching a remarkable 75% survival rate in murine models of melanoma [69]. Finally, in addition to these successful tests in preclinical melanoma models, DC-recruiting and programming PLG scaffolds also showed therapeutic activity in rat glioma models [67, 68] and mouse lung carcinoma [64].

The translation of this approach to the clinical context is presently being evaluated in a phase I clinical trial (NCT01753089) for the treatment of stage IV metastatic melanoma. It is an open-label interventional study designed to address the safety and feasibility of developing and implanting DC activating scaffolds incorporating autologous melanoma cell lysates in patients with metastatic melanoma. Additionally, as secondary objectives, the study aims to address the immune response, tumor regression and survival. This vaccine, named WDVAX, is composed of PLGA polymer and includes clinical grade GM-CSF, autologous tumor cell lysate and CpG-ODN as a DC maturation agent. The structure is implanted surgically on the patient’s arm, leg or torso by cutting a small incision into the skin and sliding it into the “pocket” created between the upper layer of the skin and the tissue underneath.

Regarding the clinical trial structure, enrollment consists of 23 patients who will receive 4 scaffolds by implantation, with skin biopsy being performed after the last vaccine. The study is divided into 3 cohorts of 3–5 patients, with each one being evaluated in a dose escalation schema, based upon the intervals between scaffold implantation at separate sites: in cohort 1, the devices are implanted monthly; in cohort 2, the implantation is performed every 3 weeks; in cohort 3, the procedure changes every 2 weeks. CT scan and/or MRI exams are performed to assess the tumor at 3 time points: before the vaccine procedure starts, halfway through the vaccination schedule and 1 month after completion of all 4 vaccines. Finally, the exam will be repeated every 3 months after the end of the protocol. The clinical study is ongoing, with results expected to be out in 2020.

#### Injectable structures

The concept of DC-recruiting structures was then expanded to other biomaterials, such as hydrogels [70, 71, 73, 79–81], mesoporous silica rods (MSRs) [74] and gelatin [82]. Hydrogel scaffolds have been applied in the biomedical field aimed at cell encapsulation in tissue engineering [83] and for controlled and sustained delivery of drugs [84–87], including therapeutic peptide and proteins [88]. Regarding DC programing platforms, hydrogel-based scaffolds offer the advantage of being deliverable through conventional needle-syringe injection, minimizing the risks and invasiveness associated with surgically implantable structures. Alginate or gelatin hydrogels developed for this purpose are normally obtained by cryogelation [80, 82]. This technique allows for the development of cryogels with considerably larger interconnected pores [89–93] and augmented mechanical stability [90] when compared to hydrogels obtained by other approaches. Briefly, the reactants are limited to the unfrozen/semi-frozen phases, forming a crosslinked network after polymerization. The ice crystals nucleated in the aqueous phase throughout freezing form pores as they melt, creating interconnected macroporous networks. Alginate cryogel produced pore sizes of 150–200 μm, high connectivity of pores, and shape-memory. These characteristics allow them to regain initial dimensions without considerable deformation after injection. Moreover, the open pore structure confers tissue-like elasticity and creates a favorable microenvironment for cell infiltration. When loaded with GM-CSF, these alginate cryogels were reported to present an encapsulation efficiency of 89%, with 80% of the total encapsulated cytokine being released within 3 days and a complete release attained after 4 weeks [80].

**Fig. 2:**
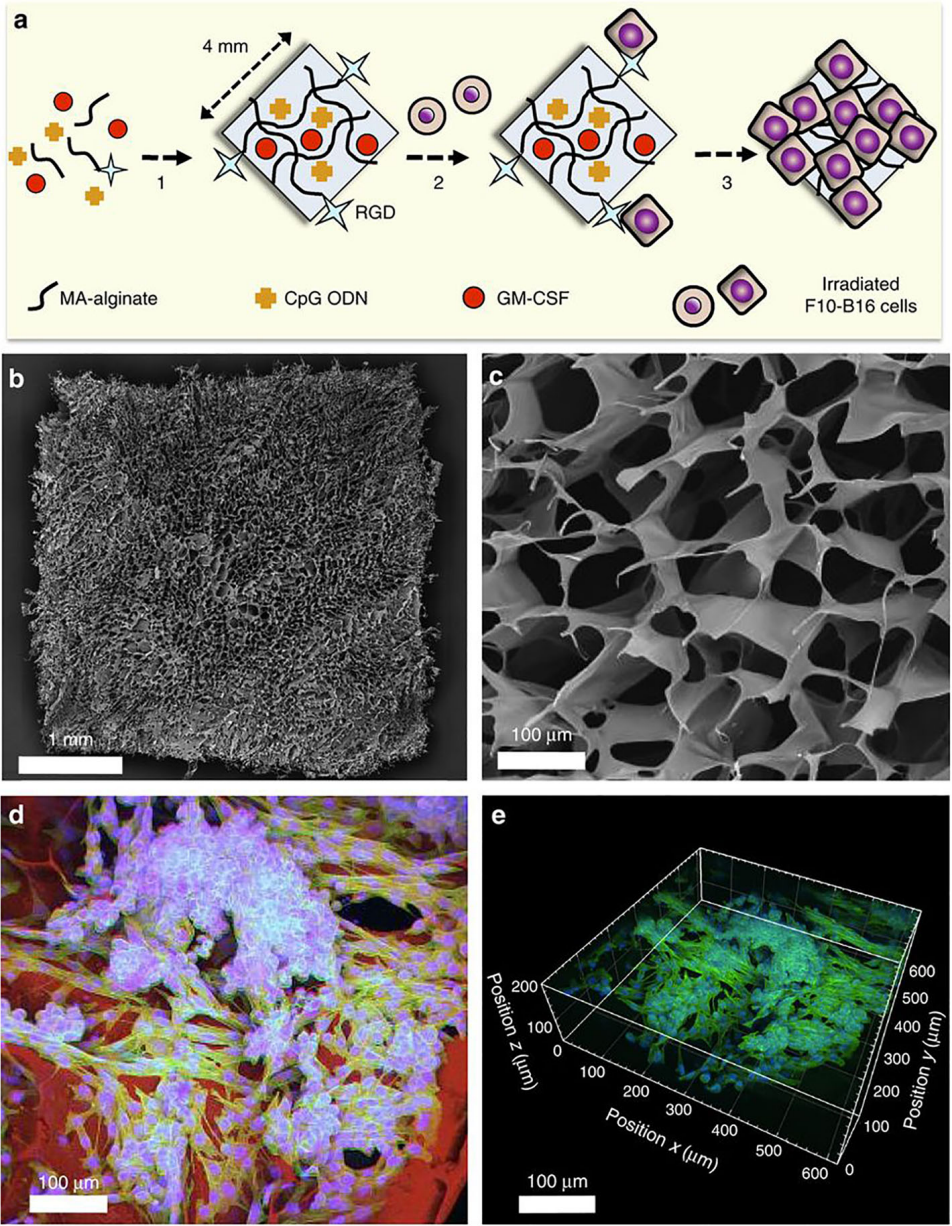
Fabrication and imaging of irradiated tumor cell-loaded cryogel sponge vaccines. **a** Preparation of an alginate-derived active vaccine containing viable irradiated B16-F10 cells for the treatment of melanoma in syngeneic C57BL/6 mice. CpG ODN (TLR9-based immune adjuvant) and GM-CSF (cytokine adjuvant)-loaded RGD-containing alginate cryogels were prepared by a cryogelation process at subzero temperature. The gels were subsequently seeded with irradiated B16-F10 melanoma cells (depicted as round-shaped cells) and incubated for 6 h (depicted as square-shaped spread cells) before animal vaccination via subcutaneous injection. **b** SEM showing homogeneous macroporous microstructure throughout the square-shaped sponge-like gel construct. **c** SEM cross-sectional image of an alginate cryogel showing the interconnected macroporous network. **d** 2D confocal micrograph displaying immobilization of irradiated B16-F10 cells on a typical RGD-containing cryogel after 6 h culture. Actin filaments in cells were visualized by staining with Alexa Fluor 488-phalloidin (green), cell nuclei were stained with DAPI (blue), and polymer walls were stained with polylysine-labeled rhodamine (red). **e** 3D reconstructed confocal fluorescence micrograph of irradiated B16-F10 cells in cryogel, depicting cell adhesion, spreading and elongation after 6 h culture. Reproduced with permission from Springer Nature, reference [71] https://www.nature.com/articles/ncomms8556 Copyright 2015

These scaffolds were preclinically tested as vaccines in several types of cancer. In mouse breast cancer models, injection of a matrix comprising live attenuated HER-2/neu-overexpressing breast cancer cells, GM-CSF and CpG-ODN resulted in the recruitment and activation of DCs followed by a robust antitumor response. The vaccine resulted in 100% survival in vaccinated mice and in a 70-fold enhancement in antibody production when compared to untreated mice [70]. In another work, alginate cryogels loaded with irradiated tumor cells and encapsulating and releasing CpG-ODN and GM-CSF in a controlled manner were tested in a mouse melanoma model **(**Fig. 2**)** [71]. This vaccine efficiently stimulated the recruitment and activation of CD8^+^ DCs, CD11^+^ DCs and pDCs. Hence, prophylactic and therapeutic protection against cancer was tested and confirmed. Specifically, potent antigen-specific T cell responses were detected, conferring long-term prophylactic protection against melanoma. With this regimen, 80% of mice survived, and importantly, of these, 100% survived a second challenge with tumor cells, indicating the induction of strong immunologic memory. When tested in a therapeutic context, two vaccination doses at days 3 and 10 post tumor establishment with B16-F10 cells strikingly resulted in complete regression of tumors in 40% of the animals [71]. Recently, the injectability of these cryogels was improved by a combination of ionic and covalent crosslinking [72]. The new scaffolds are tougher and allow for the use of a small caliber needle with no damage after injection. These improved cryogels were shown to avoid tumor development in 80% of mice injected with HER2/neu-overexpressing breast cancer cells [72].

In situ crosslinking hydrogels formed via Michael type addition of dextran vinylsulfone and tetra-thiolated polyethylene glycol were also tested as DC programming platforms [81]. These synthetic immune priming centers were loaded with CCL20 and PLGA microparticles carrying IL-10 siRNA and plasmid DNA antigen. They were shown to degrade within 2 to 7 days and to release the chemokine in a sustained manner, which resulted in up to 8-fold more DCs attracted in vivo compared to blank hydrogels [73]. Recruited DCs phagocytose microparticles and mature as observed by strong expression of CD40 and CD86. The prophylactic efficacy of these platforms was examined in mice challenged with lymphoma cells. After three immunizations separated by 14 days, animals were inoculated with lethal doses of A20-tumor cells and survived until all negative control group mice (PBS-injected) died. Vaccination resulted in a substantial enhancement in both parameters: 43 days median survival and 40% survival in immunized mice vs 32 days median survival and 0% survival in PBS group. The effect was attributed to DC-induced stimulation of potent Th1 and CTL antitumor responses [73].

MSRs are another type of biomaterial that has been tested as the core of DC programming scaffold vaccines [74]. Synthetic amorphous silica is characterized by great biocompatibility [94, 95] and safety [96] and, due to high pore volume and wide surface area, is frequently used as a carrier in controlled drug release devices [97, 98]. The DC programming scaffolds based on MSRs are synthesized with a specific hexagonal mesoporous structure via a silica sol-gel reaction in the presence of pore-directing agents [99–101]. The formed nanopores provide a high surface area for payload adsorption and surface modification [74, 102]. These MSRs spontaneously assemble in situ after injection, forming configurations with interparticle spaces that allow cell infiltration [74]. In in vitro studies, MSRs loaded with ovalbumin (OVA), CpG-ODN and GM-CSF demonstrated continuous release of the cytokine and of the TLR3 agonist during long periods. In vivo, the scaffolds increased the persistence of OVA antigen when compared to a soluble bolus and recruited large numbers of CD11c^+^ DCs, B220^+^ B cells, and CD14^+^ monocytes to the site of injection [74]. The vaccine induced potent Th1 and Th2 immune responses and antigen-specific CD8^+^ T cells, causing a significant tumor growth delay in mice subcutaneously challenged with EG7-OVA lymphoma cells [74]. The physicochemical properties of MSRs render these platforms highly tunable through modification of surface chemistry. Accordingly, diverse poly(ethylene glycol) (PEG) modifications were shown to considerably augment DC maturation and in vitro production of IL-1β as well as to boost innate immune cell infiltration in vivo [102].

## Future perspectives and concluding remarks

In recent years, biomaterial-based injectable or implantable scaffolds designed to recruit provide antigens and maturation signals to endogenous DCs have emerged as an exciting and elegant approach to elicit antitumor responses. These biomaterial-based DC programming platforms presented very promising preclinical results against several types of cancer, and the technology is expected to transition to the clinic. Accordingly, this approach is now being tested in a phase I trial in metastatic melanoma patients (WDVAX vaccine, trial NCT01753089).

The next challenge in this field will be the design of scaffolds to recruit specific DC subpopulations with superior cross-priming abilities, such as Langerhans cells and cDC1 cells (CD141^+^ CLEC9A + XCR1^+^) [103–105]. This would be achievable by loading the structures with more selective chemotactic agents: CX3CL1, CCL2 and CCL7 for Langerhans cells or XCL1/XCL2 for cDC1. The cDC1 subpopulation, apart from its exquisite cross-presenting capacity, is of particular interest because it was shown to produce, upon TLR3 engagement, IL-12p70 and IL-15, cytokines with important roles in adequate Th1 polarization and CTL and NK cell activation [106]. Moreover, given that the XCR1 ligands are selectively expressed in NK and CD8^+^ T cells, the crosstalk of these cells with cDC1 is facilitated, which is expected to result in superior antitumor immunity [107]. In fact, several preclinical studies have demonstrated that targeting antigens to Xcr1^+^CD8α DCs (mice equivalent to human cDC1) induces strong and potent antitumor responses [108, 109]. The fast-growing field of biomaterials continuously provides new technological advances, allowing the establishment of more efficient and controllable long-term release of the selected chemotactic agents. A clear example of this is the recent development of injectable lactic/glycolic copolymer microparticles functioning as pulsatile drug-delivery systems with controlled release from a few days up to 2 months [110].

Another highly desirable improvement for this vaccine technology is the loading of DCs with neoantigens encompassing individual patient tumor mutational heterogeneity. Identifying and targeting patient-specific neoantigens is considered a key feature for the development of next-generation immunotherapies [111–113]. Two seminal studies demonstrated the feasibility, safety, and immunogenicity of vaccines consisting of direct injection of melanoma–related neoantigens, either as mRNA (NCT02035956) [114] or as synthetic long peptides (NCT01970358) [115]. These works paved the way in this highly promising area, currently with more than 70 clinical trials testing neoantigen immunization. However, the definition of an optimal delivery strategy to target neoantigens to professional antigen-presenting cells to elicit potent antitumor CTL responses remains a challenge [116]. Recently, neo-epitope-loaded DCs were tested in a small phase I trial carried out on patients with advanced melanoma (NCT00683670). This vaccination approach consisted of autologous ex vivo-differentiated DCs loaded with gp100-derived peptides and seven patient-specific neoantigens. The study reported a robust induction of neoantigen-specific CD8^+^ T cells as early as 2 weeks after vaccination and the detection of memory T cells up to 4 months after the final dose [117].

Regarding biomaterial-assisted delivery of neoantigens, the existing data are extremely promising, although still only coming from preclinical studies. In one of these works, synthetic high density lipoprotein (sHDL) nanodiscs were shown to markedly improve neoantigen/CpG co-delivery to lymphoid organs and to sustain antigen presentation on DCs [118]. When tested in a murine MC38 colon carcinoma model, the sHDL structures generated a 47-fold greater frequency of neoantigen-specific CTLs when compared with the soluble neoantigen+CpG immunization. This resulted in substantially slowed tumor growth and, when combined with anti PD-1 treatment, led to complete tumor regression in 88% of tested mice, compared with only 25% observed in the soluble neoantigen+CpG + anti PD-1 treated group [118]. In another exciting work, self-assembled intertwining DNA-RNA nanocapsules (iDR-NCs) were shown to efficiently deliver CpGs, *Stat3* short hairpin RNA, and the MC38 tumor neoantigen Adpgk into APCs. Immunization of C57BL/6 mice with iDR-NC/Adpgk nanovaccines elicited an 8-fold increase in specific CTLs relative to soluble CpG + Adpgk, induced immunological memory and significantly inhibited the progression of colorectal tumors [119]. Finally, mesoporous silica micro-rods combined with polyethyleneimine (PEI), the MSR-PEI vaccine, were also recently tested as a platform for neoantigen delivery [120]. A single immunization with MSR-PEI containing a pool of B16F10 or CT26 neoantigens significantly increased IFNγ^+^, TNFα^+^ and Granzyme B^+^ TILs. Furthermore, the vaccine controlled tumor growth and eradicated established lung metastases of respective tumors, synergizing with anti-CTLA4 therapy.

The combination of biomaterials-based platforms for in situ programming of DCs with other immunotherapies is also expected to contribute to more robust and effective antitumor immune responses. Due to their clear clinical effectiveness, immune checkpoint inhibitors are promising candidates for these associations [121, 122]. These combinatory therapeutic regimens will tackle multiple aspects of the tumor immunoediting process: the vaccine boosts the elimination phase by eliciting and expanding effector immune cells, while checkpoint inhibitors block major tumor escape mechanisms. In fact, numerous clinical trials focused on DC vaccines targeting cancer are currently testing their association with checkpoint inhibitors [123]. Interestingly, while sipuleucel-T presented moderate clinical outputs as a monotherapy, early observations from recent trials investigating its combination with atezolizumab (Anti-PD-L1) (NCT03024216) or ipilimumab (NCT01804465) show very promising results [124]. Hence, it is also expected that the number of studies exploring the combination of biomaterial-based DC programming vaccines with immune checkpoint inhibitors, such as PDL-1, PD-1 and CTLA-4 mAbs, will strongly increase in the next few years. Indeed, PLG scaffolds combined with anti CTLA-4 or anti PD-1 antibodies were already tested and reported to elicit strong CTL activity and tumor elimination in murine models of melanoma [69]. Follow-up studies of this strategy for a consequent translation to clinical trials are needed, allowing the development of novel and more thrilling paths in cancer immunotherapy.

## Data Availability

Not applicable.

## *Abbreviations*

APC: Antigen-presenting cell;
CAR: Chimeric antigen receptor
CCL19: Chemokine ligand 19
cDC1: Conventional type 1 dendritic cells
CpG-ODN: CpG oligonucleotide
CT: Computed tomography
CTL: Cytotoxic T-lymphocyte
CTLA-4: Cytotoxic T-lymphocyte antigen 4
CXCR3: Chemokine receptor CXCR3
DC: Dendritic cell
EVA: Ethylene-vinyl-acetate
FDA: Food and drug administration
GM-CSF: Granulocyte-macrophage colony-stimulating factor
GMP: Good manufacturing practices
HLA: Human leucocyte antigens
IFN-γ: Interferon gamma
IL: Interleukin
LC: Langerhans cell
LLC: Lewis lung carcinoma
mAb: Monoclonal antibody
MHC: Major histocompatibility complex
mPEG-PLGA: monomethoxypoly(ethylene glycol)-*co*-poly(lactic-*co*-glycolic acid)
MPLA: Monophosphoryl lipid A
MRI: Magnetic resonance imaging
MSR: Mesoporous silica rod
NK: Natural killer
OVA: Ovalbumin
PBMCs: Peripheral blood mononuclear cells
pDC: plasmacytoid dendritic cell
PD-L1: Programmed cell death ligand 1
PEG: Poly(ethylene glycol)
PLG: Poly(lactide-co-glycolide)
Poly-I:C: Polyinosinic:polycytidylic acid
TAA: Tumor-associated antigens
Th1: T helper cell type 1
Th2: T helper cell type 2
TIL: Tumor-infiltrating lymphocytes
TLR: Toll-like receptor
TNF: Tumor necrosis factor
